# Supplementary material for: Identifying topological corner states in two-dimensional metal-organic frameworks
Source: Nat Commun. 2023 Nov 4;14:7092. doi: 10.1038/s41467-023-42884-1 (PMC10625601; doi:10.1038/s41467-023-42884-1)
Supplement: Supplementary file 1 — Supplementary Information [file 41467_2023_42884_MOESM1_ESM.pdf]

Supplementary Materials

Identifying Topological Corner States in Two-Dimensional Metal-Organic Frameworks

Tianyi Hu[1], Weiliang Zhong[2], Tingfeng Zhang[1], Weihua Wang[2*], and Z. F. Wang[1,3*]

[1]Hefei National Research Center for Physical Sciences at the Microscale, CAS Key Laboratory of Strongly-Coupled Quantum Matter Physics, Department of Physics, University of Science and Technology of China, Hefei, Anhui 230026, China

[2]Beijing National Laboratory for Condensed Matter Physics, Institute of Physics, Chinese Academy of Sciences, Beijing 100190, China

[3]Hefei National Laboratory, University of Science and Technology of China, Hefei, Anhui 230088, China

*Correspondence to: weihuawang@iphy.ac.cn; zfwang15@ustc.edu.cn

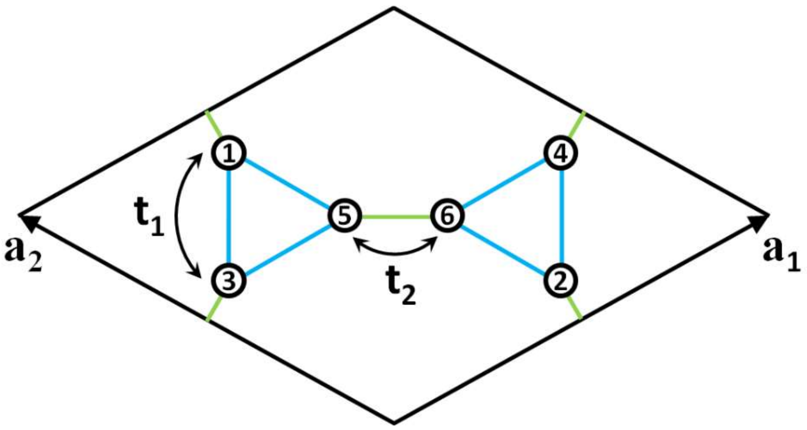

Supplementary Figure 1. Star lattice. Definition of hopping parameter ($t_{1,2}$), lattice site (1-6) and lattice vector ($\mathbf{a}_{1,2}$) in star lattice.

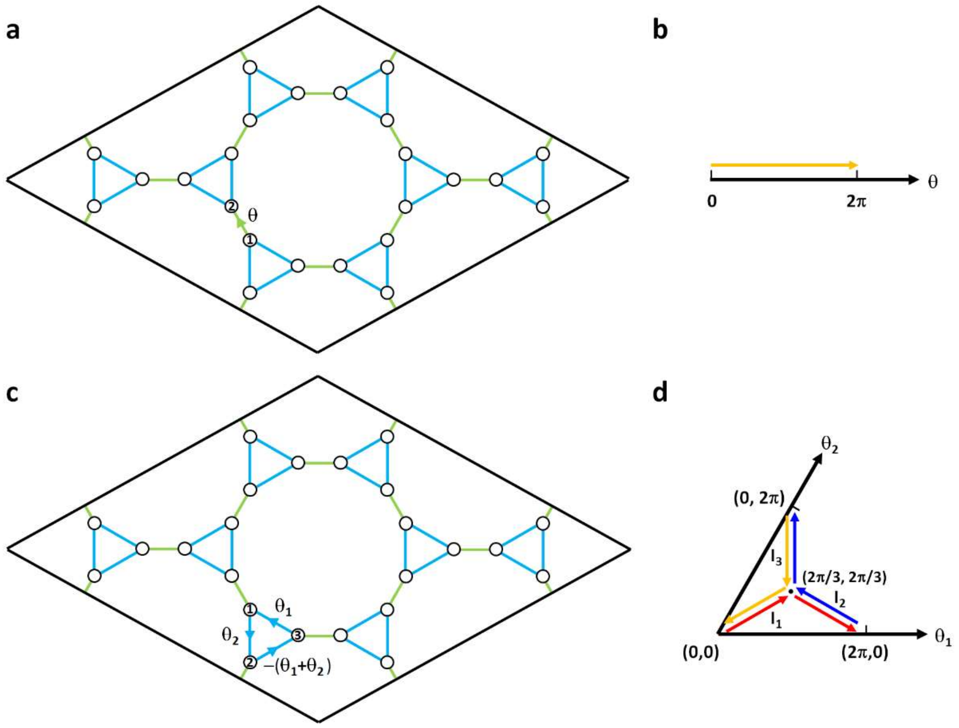

Supplementary Figure 2. Supercell Berry phase calculation. a, The 2×2 supercell for calculating the $\mathbb{Z}_2$ Berry phase. The local twisted TB Hamiltonian is introduced in one dimer between lattice site 1 and 2 with a hopping phase of θ. **b,** Schematic path of contour integral in Eq. 5 in parameter space of θ. **c,** The 2×2 supercell for calculating the $\mathbb{Z}_3$ Berry phase. The local twisted TB Hamiltonian is introduced in one trimer among lattice site 1, 2 and 3 with three hopping phases of θ_1, θ_2, and $-(\theta_1+\theta_2)$. **d,** Schematic paths $I_{1,2,3}$ of contour integral in Eq. 7 in parameter space of θ_1 and θ_2.

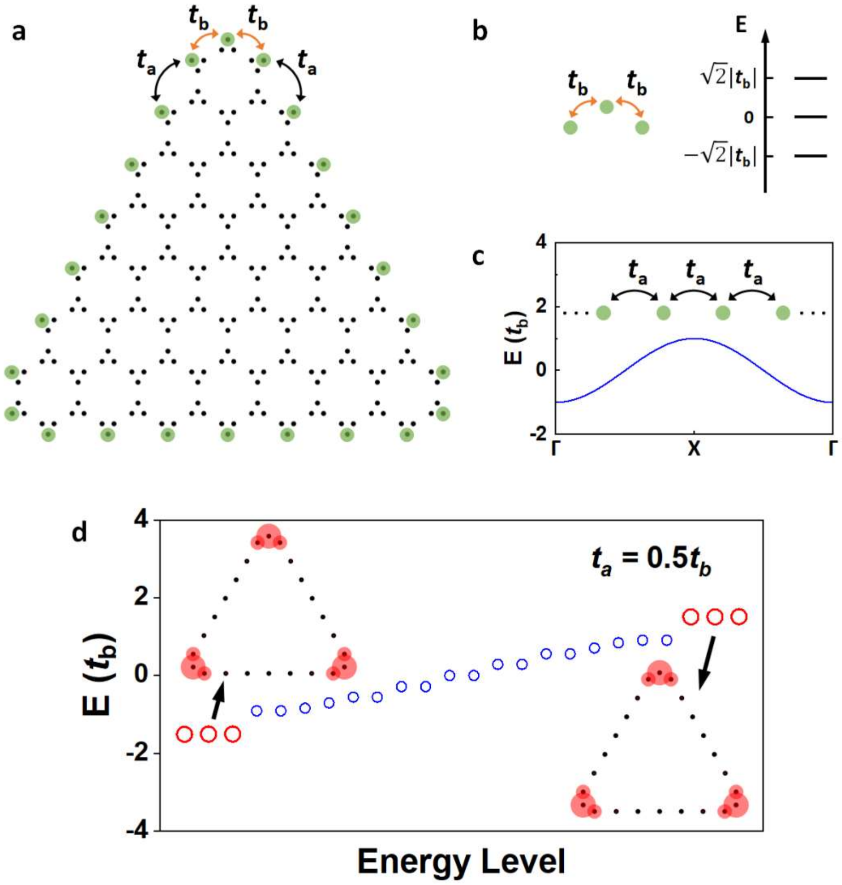

Supplementary Figure 3. Effective edge hopping. a, Effective boundary hopping for broken dimers (highlighted by green dots) in triangular cluster with Edge-A termination. Along the edge, the hopping is defined as t_a. At the corner, the hopping is defined as t_b. **b,** The effective boundary hopping at the corner generates three energy levels, siting at 0 and $\pm\sqrt{2}|t_b|$. **c,** The effective boundary hopping along the edge, forming a monoatomic wire (inset), generates a one-dimensional band for edge states. **d,** The discrete energy levels of triangular cluster constructed from monoatomic wires with three corners. The insets show the spatial distribution of corner states. The circle size denotes the weighting factor of corner states.

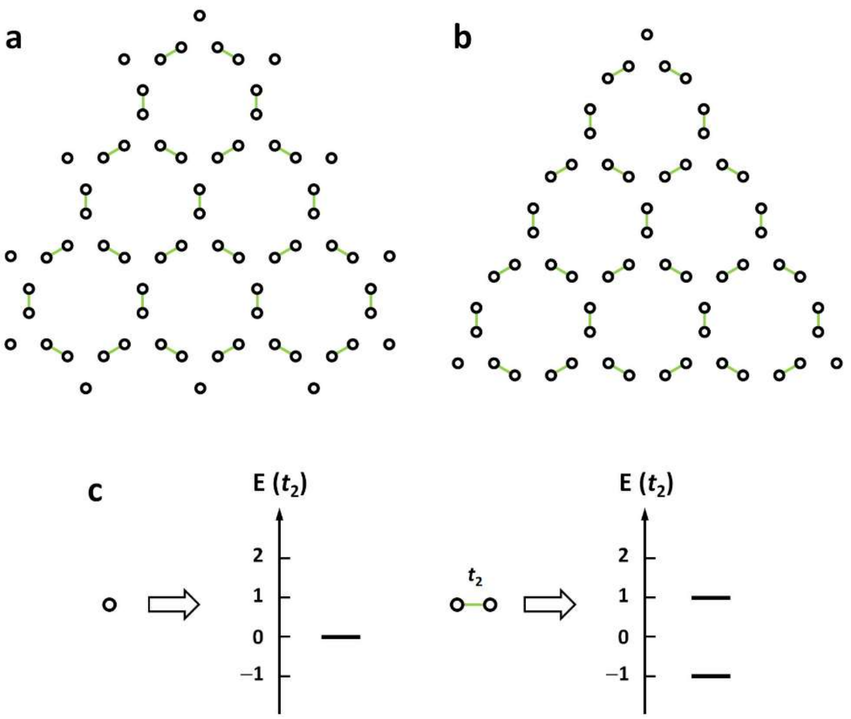

Supplementary Figure 4. **Triangular cluster in decoupled limit. a,** Triangular cluster of star lattice with Edge-A termination in decoupled limit. The bulk, edge and corner are constructed by dimer, monomer, and monomer, respectively. **b,** Triangular cluster of star lattice with Edge-B termination in decoupled limit. The bulk, edge and corner are constructed by dimer, dimer, and monomer, respectively. The sold lines in **a,b** denote the hopping of t_2. **c,** Energy levels of the monomer and dimer. For star lattice with Edge-A termination, the edge states are in the gap of bulk states. Therefore, the edge states are distinguishable with bulk states. For star lattice with Edge-B termination, the edge and bulk states have the same energy levels. Therefore, the edge states are indistinguishable with bulk states.

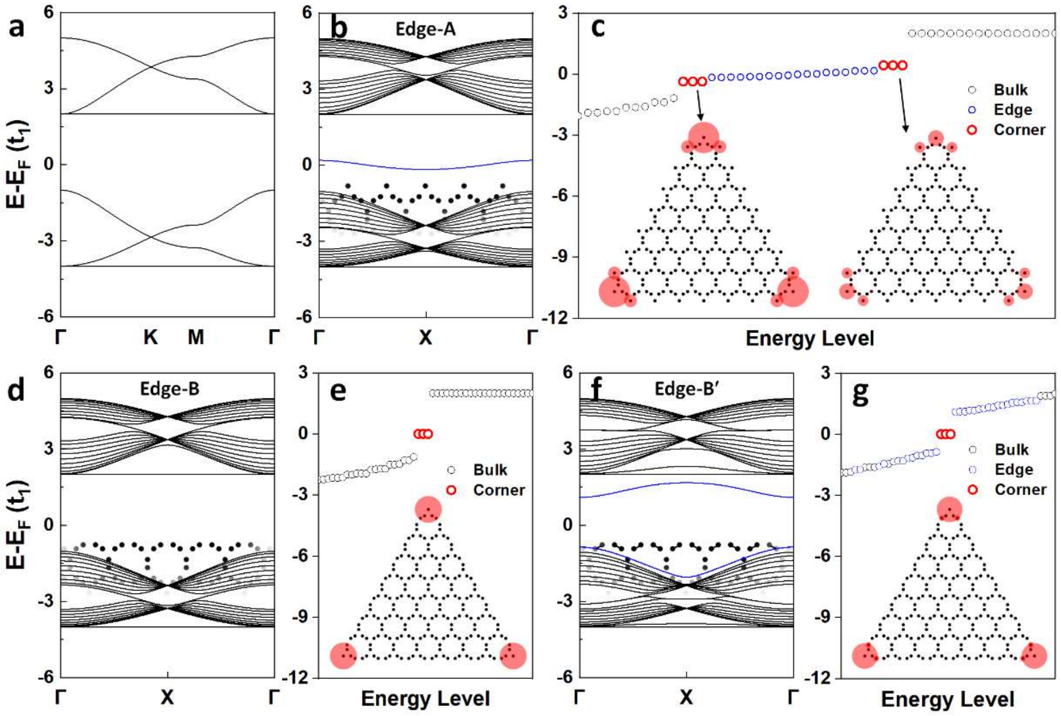

Supplementary Figure 5. Topological corner states in Type-II bands. a, Type-II bands with Fermi-level between two Kagome-bands, corresponding to 1/2 filling. **b, d** and **f** Ribbon band structures with Edge-A, Edge-B and Edge-B′ termination, respectively. The inset shows the shape of edge structure. Edge-B′ is a slightly modified Edge-B with t_2=2.1t_1 in outmost dimers, as denoted by solid lines in inset of **f**. **c, e** and **g** Discrete energy-levels of triangular cluster with Edge-A, Edge-B and Edge-B′ termination, respectively. The inset shows the spatial distribution of corner states. The circle size denotes weighting factor of corner states. The black, blue and red color in **b-g** denotes bulk, edge and corner state, respectively. The hopping parameter is set to t_2=3t_1. Each corner state in **e** and **g** holds a fractional charge of e/2.

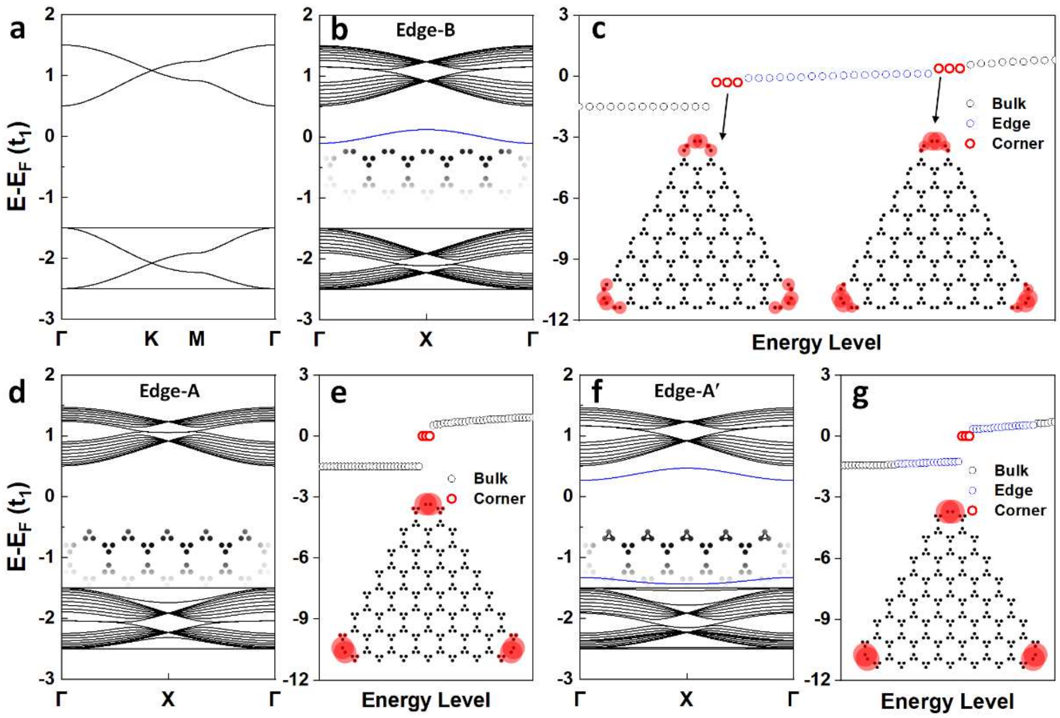

Supplementary Figure 6. Topological corner states in Type-IV bands. a, Type-IV bands with Fermi-level between Dirac-band and four-band, corresponding to 2/3 filling. **b, d** and **f** Ribbon band structures with Edge-B, Edge-A and Edge-A′ termination, respectively. The inset shows the shape of edge structure. Edge-A′ is a slightly modified Edge-A with t_2=0.35t_1 in outmost trimers, as denoted by solid lines in inset of **f**. **c, e** and **g** Discrete energy-levels of triangular cluster with Edge-B, Edge-A and Edge-A′ termination, respectively. The inset shows the spatial distribution of corner states. The circle size denotes weighting factor of corner states. The black, blue and red color in **b**-**g** denotes bulk, edge and corner state, respectively. The hopping parameter is set to t_2=0.5t_1. Each corner state in **e** and **g** holds a fractional charge of e/3.

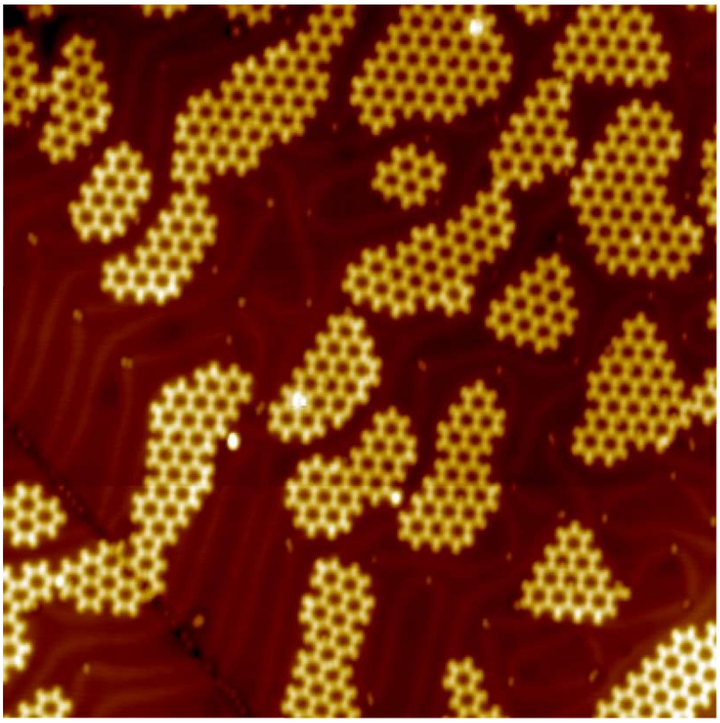

Supplementary Figure 7. STM image of $Ni_3(HITP)_2$. Large-scale STM image of monolayer $Ni_3(HITP)_2$ frameworks on Au(111) substrate (−1.0 V, 10 pA, 80 nm × 80 nm), showing the formation of many triangular shaped clusters.

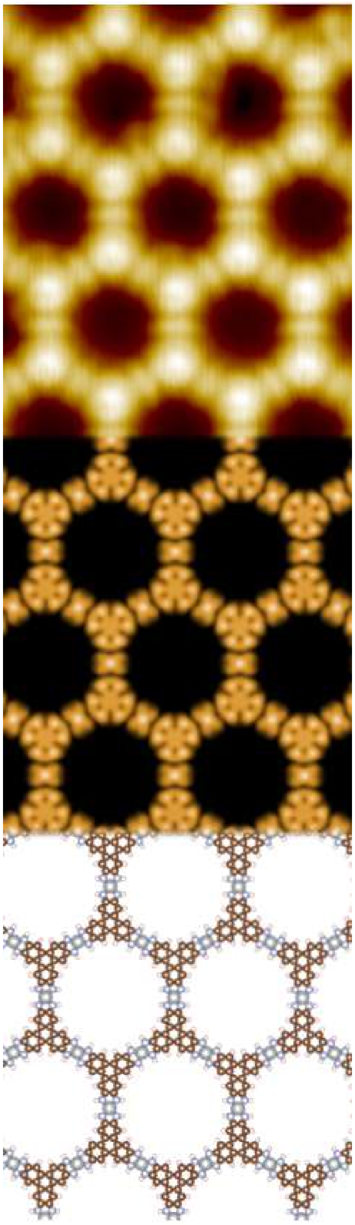

Supplementary Figure 8. **Comparison between experimental and theoretical STM image.** Top panel: Zoomed-in STM image of $Ni_3(HITP)_2$, scanned at −0.9 V and 300 pA. Middle panel: Simulated STM image of $Ni_3(HITP)_2$ within 0.9 eV below the Fermi level. Bottom panel: Atomic structures of $Ni_3(HITP)_2$. The simulated STM image shows good agreement with the experimental image.

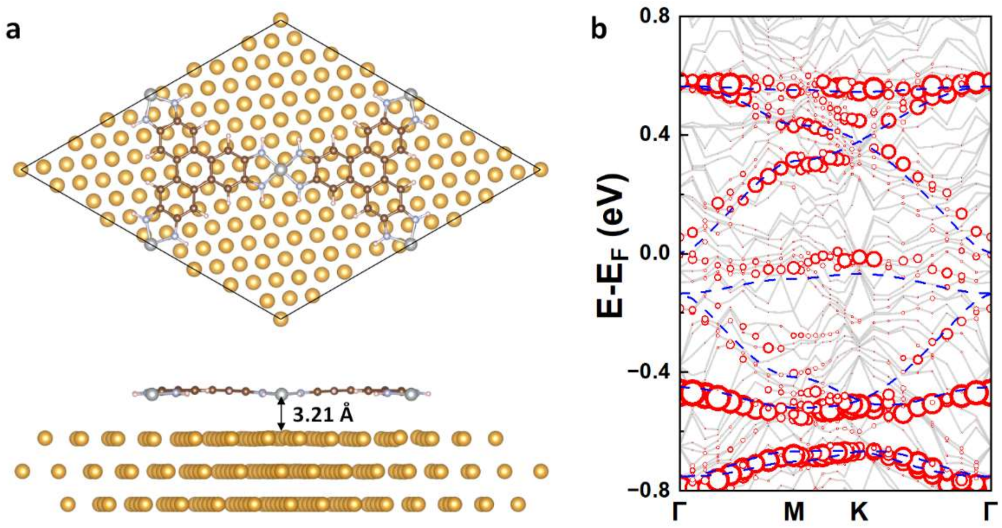

Supplementary Figure 9. Band structures of $Ni_3(HITP)_2$/Au(111). a, Top and side views of atomic structures of $Ni_3(HITP)_2$/Au(111). The vertical distance between $Ni_3(HITP)_2$ and Au(111) substrate is 3.21 Å. **b,** Projected band structures of $Ni_3(HITP)_2$/Au(111). The grey lines are total bands. The red circles are projected bands of $Ni_3(HITP)_2$. The circle size denotes weighting factor of $Ni_3(HITP)_2$. The blue dashed lines are freestanding bands of $Ni_3(HITP)_2$. Obviously, the feature of Type-I bands is kept by including substrate, showing the weak coupling between $Ni_3(HITP)_2$ and Au(111) substrate.

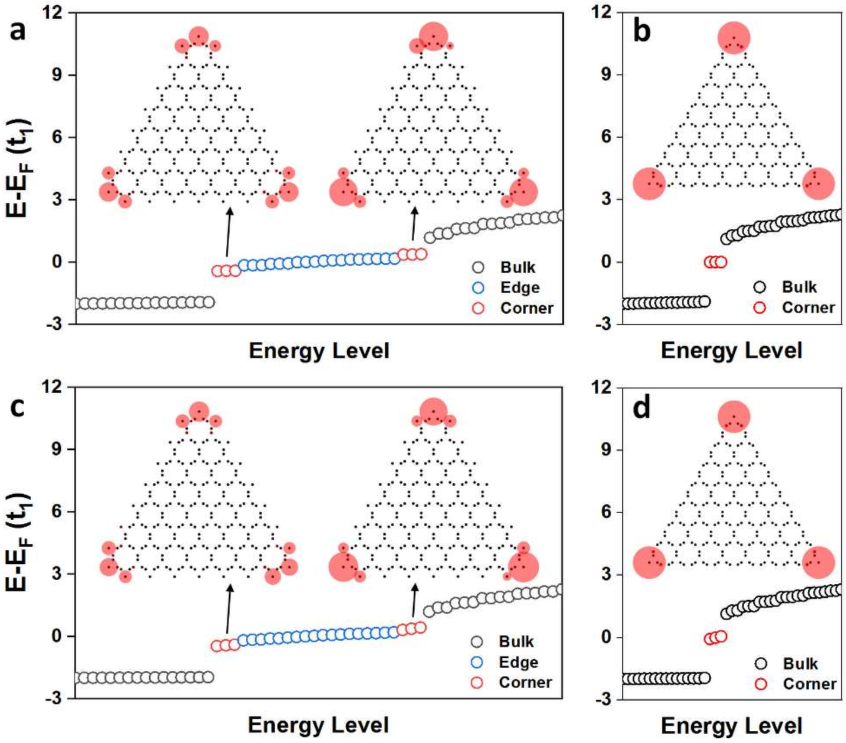

Supplementary Figure 10. Corner states under the random perturbation. a,b, Discrete energy-levels of triangular clusters for Type-I bands with Edge-A and Edge-B termination, respectively. The random hopping is added to t_1 and t_2 with $\delta t \in [-0.1t_1, 0.1t_1]$. **c,d,** Discrete energy-levels of triangular clusters for Type-I bands with Edge-A and Edge-B termination, respectively. The random onsite energy is added with $\delta\varepsilon \in [-0.1t_1, 0.1t_1]$. The inset shows the spatial distribution of corner states. The circle size denotes the weighting factor of the corner states. The black, blue and red colors denote the bulk, edge and corner states, respectively. The hopping parameter is set to $t_2=3t_1$. Including the small random perturbation, the overall feature is the same to that without perturbation.

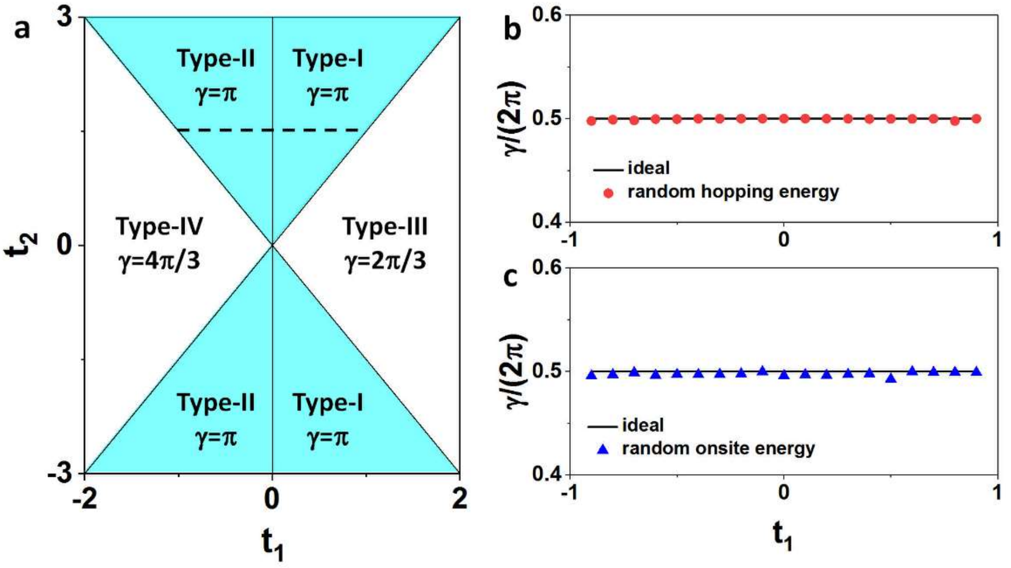

Supplementary Figure 11. Berry phase under the random perturbation. a, Ideal topological phase diagram vs $t_{1,2}$ classified by Berry phase γ. Type-I, II, III, IV denote four different bands. **b,** Berry phase γ along the dashed line in **a** by including random hopping energy $\delta t \in [-0.1, 0.1]$. **c,** Berry phase γ along the dashed line in **a** by including random onsite energy $\delta\varepsilon \in [-0.1, 0.1]$. As a comparison, the ideal quantized Berry phase without perturbation (solid line) is also plotted in **b** and **c**. Clearly, the Berry phase γ just exhibits a small fluctuation around its quantized value, so the nontrivial higher-order topology is still maintained under the small random perturbation.

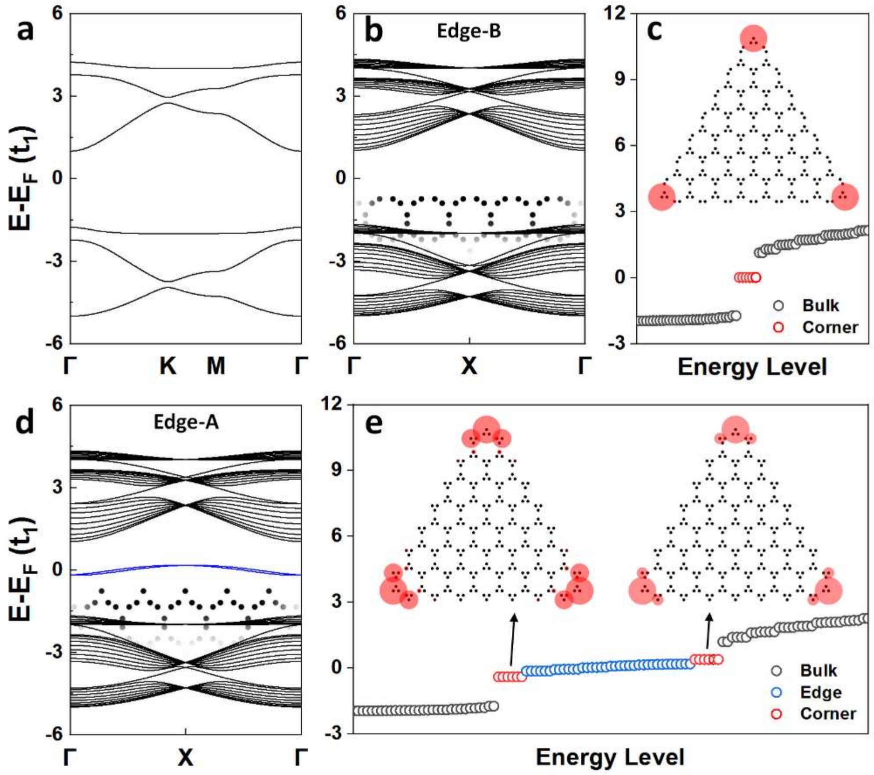

Supplementary Figure 12. Robustness of the higher-order topology with SOC. a, Type-I bands with intrinsic SOC. **b,d,** Ribbon band structures with Edge-B and Edge-A termination, respectively. The insets show the shape of the edge termination. **c,e,** Discrete energy-levels of the triangular cluster with Edge-B and Edge-A termination, respectively. The insets show the spatial distribution of corner states. The circle size represents the weighting factor of the corner states. The black, blue and red colors in **b-e** denote the bulk, edge and corner states, respectively. The hopping parameter is set to t_2=3t_1, and the intensity of SOC is set to λ=0.1t_1. Including the intrinsic SOC, the number of the corner states is doubled, but the overall feature is the same to that without SOC.
